# Selective syntheses of thick and thin nanosheets based on correlation between thickness and lateral-size distribution

**DOI:** 10.1016/j.isci.2022.104933

**Published:** 2022-08-24

**Authors:** Yuri Haraguchi, Hiroaki Imai, Yuya Oaki

**Affiliations:** 1 Department of Applied Chemistry, Faculty of Science and Technology, Keio University, 3-14-1 Hiyoshi, Kohoku-ku, Yokohama 223-8522, Japan

**Keywords:** Materials science, Materials synthesis, Nanomaterials

## Abstract

Exfoliation of layered materials, a typical route to obtain 2D materials, is not easily controlled because of the unpredictable downsizing processes. In particular, the thickness control remains as a complex challenge. Here, we found a correlation between the thickness and lateral size distribution of the exfoliated nanosheets, such as transition metal oxides and graphene oxide. The layered composites of the host metal oxides and interlayer organic guests are delaminated into the surface-modified nanosheets in organic dispersion media. The exfoliation behavior varies by combination of the hosts, guests, and dispersion media. Here, we found that the thick and thin nanosheets were obtained on the monodispersed and polydispersed conditions, respectively. The selective syntheses of the thick and thin nanosheets were achieved using a prediction model of the lateral size distribution. The correlation between the thickness and lateral size distribution can be applied to thickness-selective syntheses of 2D materials.

## Highlights

• Surface-modified nanosheets are obtained by exfoliation of layered composites
• Thickness of 2D materials has a correlation with the lateral size distribution
• Thick and thin nanosheets are selectively synthesized under the predicted conditions
• A prediction model of lateral size distribution is applied to the selective syntheses

## Graphical abstract

**Figure:**
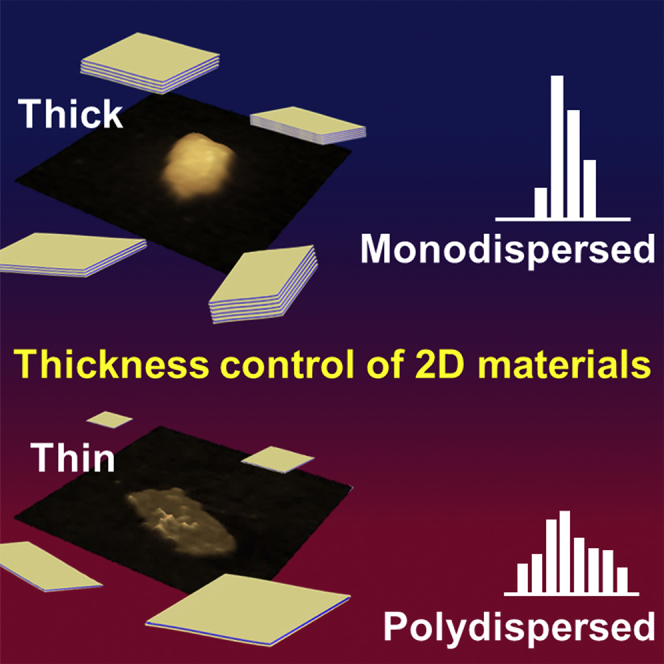

## Introduction

Nanosheets including monolayers and few layers are found in a variety of materials, such as graphene, transition metal dichalcogenides, hexagonal boron and carbon nitrides, black phosphorus, clays, metal oxides, metal-organic frameworks, and organic polymers (Nicolosi et al., 2013; Pumera et al., 2014; Cong et al., 2014; Ma and Sasaki, 2015; Zhuang et al., 2015; Mendoza-Sánchez and Gogotsi, 2016; Tan et al., 2017; Zhang et al., 2018; Rao and Peamoda, 2019; Timmerman et al., 2020; Li et al., 2020; Wang et al., 2020; Oaki, 2021). The characteristic properties originating from the 2D anisotropy and ultrathin nanostructures, such as high specific surface area, quantum-size effect, and flexibility, have potentials for their diverse applications (Nicolosi et al., 2013; Pumera et al., 2014; Cong et al., 2014; Ma and Sasaki, 2015; Zhuang et al., 2015; Mendoza-Sánchez and Gogotsi, 2016; Tan et al., 2017; Zhang et al., 2018; Rao and Peamoda, 2019; Timmerman et al., 2020; Li et al., 2020; Oaki, 2020, 2021; Wang et al., 2012; Ariga et al., 2018; Ganter and Lotsch, 2019; Xiong et al., 2020; Wang et al., 2020; Oaki, 2020). Pristine layered compounds are classified into the two types depending on the interlayer interaction, such as van der Waals and electrostatic interactions (Oaki, 2020). In general, 2D materials are obtained by liquid-phase exfoliation of layered compounds based on van der Waals interaction under sonication, *i*.*e*. mechanical exfoliation, triggered by shear stress. Although 2D materials are regarded as a family of recent promising nanostructures, the size control, such as the lateral size and thickness, is not easily achieved only by changes in the experimental conditions. The unpredictable downsizing processes including exfoliation and fracture in vertical and lateral directions form the nanosheets with the random sizes, respectively. In particular, the control of the thickness, *i*.*e*. the layer number, is a significant remaining challenge. If the thickness of the nanosheets is predicted and controlled, a variety of the size-dependent properties (Suk et al., 2010; 2020; Backes, 2014; ten Elshof, 2017; Rahmanian and Malekfar, 2017; Liu et al., 2017; Mori et al., 2018; Miao et al., 2019), such as bandgap energy, specific surface area, and flexibility, can be tuned for their applications.

**Figure 1:**
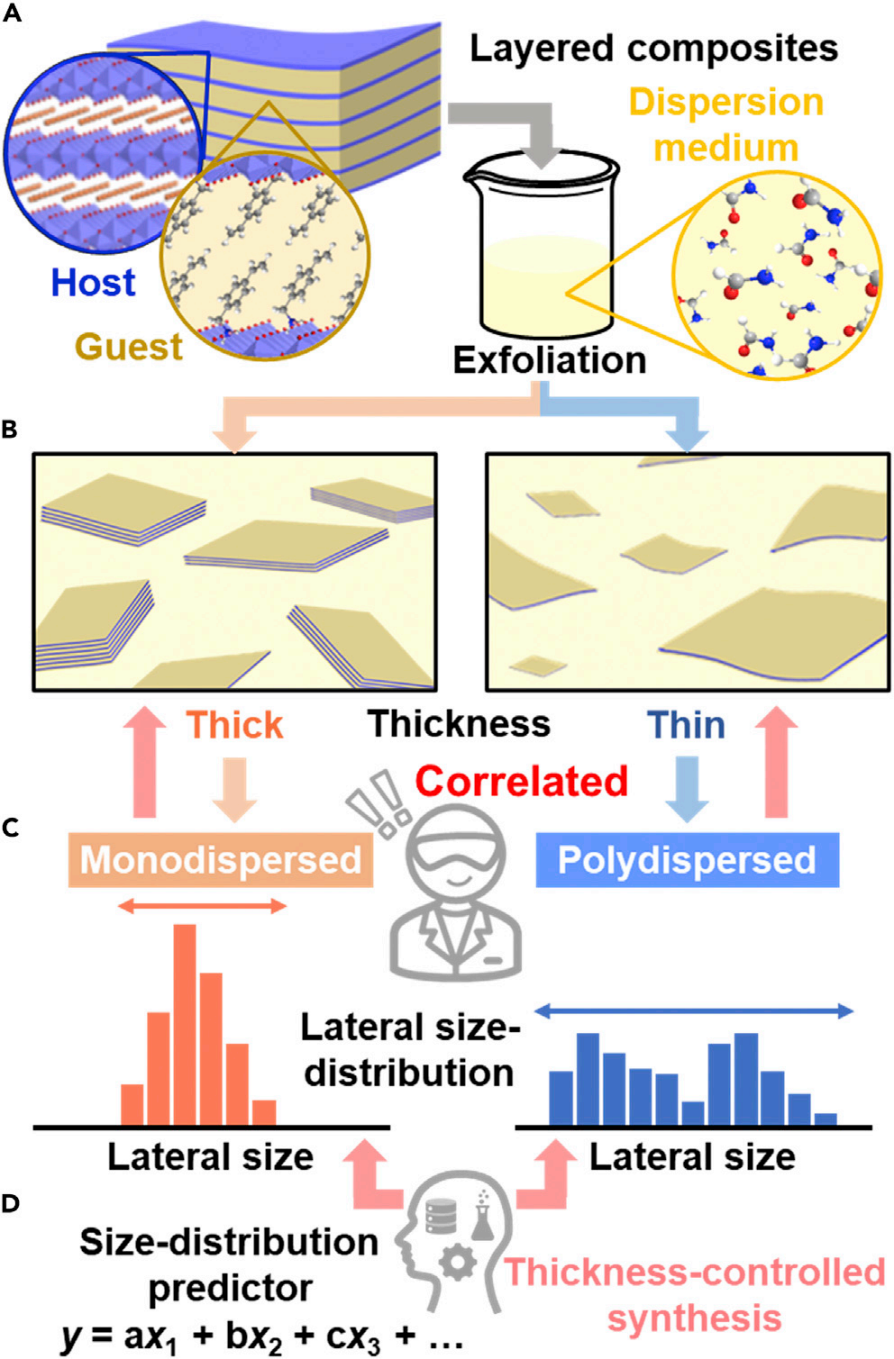
Schematic illustrations of the correlation between the thickness and lateral size distribution on the exfoliated nanosheets (A) Layered composites of host transition metal oxides and guest organic molecules and their exfoliation into the thick and thin nanosheets with the dispersion in organic media. (B) Thick and thin nanosheets with the monodispersed and polydispersed lateral size, respectively. (C) Monodispersed (left) and polydispersed (right) lateral sizes correlated with the thickness. (D) Size-distribution prediction model, constructed by an assistance of machine learning (Haraguchi et al., 2021), applicable to the thickness-selective syntheses of the nanosheets (red arrows).

In addition to the unpredictable processes, the time- and effort-consuming analytical processes deter the researchers from the study on the thickness. In general, thickness of nanosheets is measured by atomic force microscopy (AFM) and summarized in the histogram. Although the high-throughput estimation of the thickness was studied by microscopy images with an assistance of machine learning (Ni et al., 2007; Nolen et al., 2011; Li et al., 2013; Backes et al., 2016a, 2016b; Masubuchi et al., 2018; Masubuchi and Machida, 2019; Han et al., 2020), the method was applied to the limited types of the layered materials. New methods and insights are required to selective syntheses of thin and thick nanosheets. The changes in the thickness were observed depending on the experimental conditions, such as the exfoliation time and types of the applied stimuli (Miyamoto and Nakato, 2004; Varrla et al., 2015; Tayyebi et al., 2020; Qi et al., 2021; Chacham et al., 2020). The nanosheets with the specified thickness and lateral size were collected by tuning the purification conditions of the dispersion liquids during centrifugation and electrophoresis (Khan et al., 2012; Backes et al., 2016a, 2016b; Tay et al., 2018; Alzakia et al., 2020). The designed layered materials provided the nanosheets with the specified layer numbers (Miyamoto et al., 2002; Maluangnont et al., 2013; Kimura et al., 2014; Nakada et al., 2018; Lee et al., 2019; Singha Mahapatra et al., 2020). These previous works mainly focused on the effects of the experimental parameters on the thickness (Miyamoto and Nakato, 2004; Varrla et al., 2015; Tayyebi et al., 2020; Qi et al., 2021; Chacham et al., 2020; Khan et al., 2012; Backes et al., 2016a, 2016b; Tay et al., 2018; Alzakia et al., 2020; Miyamoto et al., 2002; Maluangnont et al., 2013; Kimura et al., 2014; Nakada et al., 2018; Lee et al., 2019; Singha Mahapatra et al., 2020). If the relevance of the chemical and/or structural factors to the thickness is elucidated, the selective syntheses of thick and thin nanosheets can be achieved efficiently. Here, we found the correlation between the thickness and lateral size distribution of the exfoliated nanosheets based on transition metal oxides (Figures 1A–1C). This model case can be applied to the other exfoliation systems.

Layered transition metal oxides consist of negatively charged host layers and positively charged guest ions. The layered composites are prepared by intercalation of the cationic organic guests. Our group has studied that the surface-functionalized nanosheets are obtained by exfoliation of the layered composites in organic dispersion media (Figures 1A and 1B) (Oaki, 2021; Honda et al., 2014). As the exfoliation behavior is tuned by the host-guest-medium combinations, this exfoliation system is preferable to study the factors for control of the thickness. The prediction models of the yield, lateral size, and lateral size distribution were constructed by combination of machine learning and chemical insights (Haraguchi et al., 2021; Nakada et al., 2019; Noda et al., 2020, 2021; Mizuguchi et al., 2021). The prediction models using the physicochemical parameters of the host, guest, and medium were applied to the selective syntheses in a limited number of the exfoliation experiments. However, the factors correlated with the thickness have not been found in the previous works. Our intention here is to study the factors correlated with the thickness and to demonstrate the selective syntheses of the thick and thin nanosheets. We found the relationship between the lateral size distribution and thickness (Figures 1B and 1C). Moreover, the prediction model of the size distribution was applied to selective synthesis of the thick and thin nanosheets in a limited number of the experiments (Figures 1C and 1D). The correlation can be applied to thickness control for the other layered compounds.

## Results

The precursor layered composites were prepared and characterized according to our previous works (Haraguchi et al., 2021; Honda et al., 2014; Nakada et al., 2019; Noda et al., 2020, 2021; Mizuguchi et al., 2021). The following layered metal oxides were used as the negatively charged hosts: layered titanate, manganate, and niobate. The cationic organic guests, such as alkyl amines and benzyl amines, were intercalated in the interlayer space. Cobalt hydroxide as the positively charged host accommodated the anionic guests, such as carboxylates. In addition, stacked graphene oxide (GO) was used as another layered material with a different type of interlayer interaction via van der Waals. These precursor layered materials were dispersed in organic dispersion media, such as ethanol and formamide, under mild conditions for 5 days at 60°C with stirring at 300 rpm (Figure 1A). After the unexfoliated bulky particles were removed by filtration, the dispersion liquids containing the exfoliated nanosheets were obtained.

The lateral size distribution of the exfoliated nanosheets was measured by dynamic light scattering (DLS) of the dispersion liquid (Figures 1B and 1C). DLS was used to achieve high-throughput estimation of the lateral size (Haraguchi et al., 2021; Mizuguchi et al., 2021), even though the accurate size of the anisotropic objects was not measured. The correlation of the lateral size between the DLS measurement and microscopy analysis was studied in the previous works (Haraguchi et al., 2021; Mizuguchi et al., 2021; Lotya et al., 2013; Yano et al., 2019). The parameter of the lateral size distribution was defined as the coefficient of the variation (*L*_CV_ = *σ*/*L*_ave_), where *L*_ave_ and *σ* are the average size and its standard deviation measured by DLS, respectively (Haraguchi et al., 2021). The correlation of the size distribution between the DLS analysis and TEM measurement was studied in our previous work (Haraguchi et al., 2021). The thickness (*t*) of the nanosheets measured by AFM was summarized in the histogram. As *t* is not simply compared with the different combinations of the hosts and guests, *t* is converted to the layer numbers (*N*) on the assumption that the interlayer distance estimated from the XRD analysis corresponds to the thickness of the monolayer (Figure S1).

### Correlation between lateral size distribution and thickness

**Figure 2:**
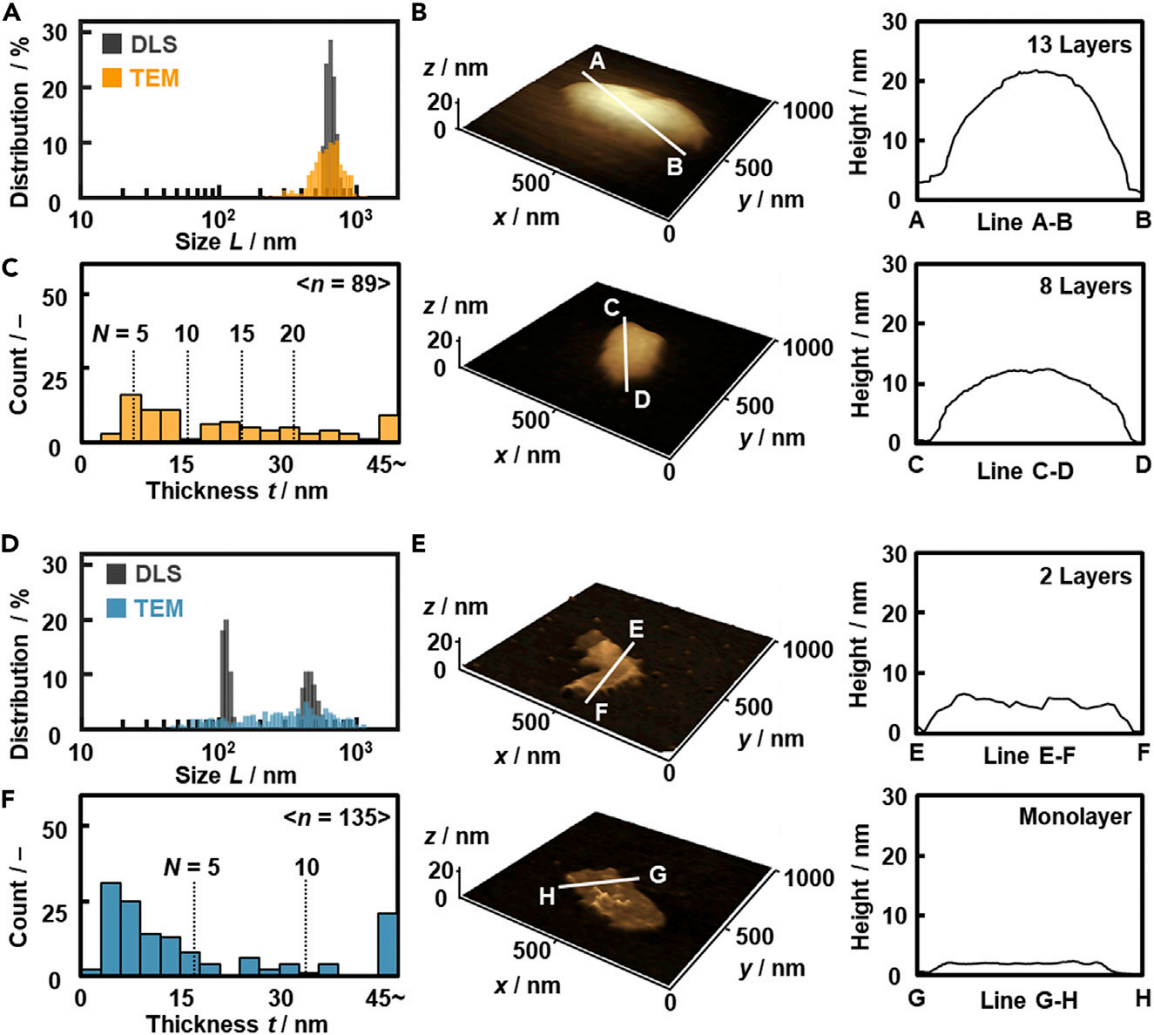
Lateral size distribution and thickness of the (NH_2_-BA)-titanate nanosheets in 2-butanol (A–C) and (C_18_-NH_2_)-titanate nanosheets in benzaldehyde (D–F) (A–D) Lateral size distribution estimated from DLS (black) and TEM (colored) analyses. (B–E) AFM images and their height profiles. (C–F) Histogram of the thickness based on the AFM images.

The thick and thin nanosheets had the monodispersed and polydispersed size distributions, respectively (Figure 2). According to our previous work (Haraguchi et al., 2021), layered titanate with the intercalation of 4-aminobenzylamine (NH_2_-BA) and octadecylamine (C_18_-NH_2_) provided the (NH_2_-BA)-titanate nanosheets in 2-butanol and (C_18_-NH_2_)-titanate nanosheets in benzaldehyde, respectively. The lateral size distribution was summarized in Figures 2A and 2D. The (NH_2_-BA)-titanate and (C_18_-NH_2_)-titanate nanosheets showed the monodispersity with *L*_CV_ = 0.077 and polydispersity with *L*_CV_ = 0.627 using DLS analysis, respectively (Figures 2A and 2D). The anisotropic nanosheets were observed on the AFM images (Figures 2B and 2E). The *L*_CV_ values based on the transmission electron microscopy (TEM) images (*L*_CV,TEM_) were *L*_CV,TEM_ = 0.246 for the (NH_2_-BA)-titanate nanosheets and 0.679 for the (C_18_-NH_2_)-titanate nanosheets (Figures 2A and 2D). In general, DLS analysis assumes colloidal dispersion of three-dimensionally isotropic spheres. On the other hand, the nanosheets have 2D anisotropic shape. The size of the nanosheets is approximated to the diameter of the circumscribed sphere in DLS analysis. The lateral size of the nanosheets corresponds to be the length of the longitudinal axis. DLS analysis shows the approximated average size in the colloidal state. On the other hand, TEM images show a limited number of the nanosheets in the local area in the dried state. The *L*_CV_ values estimated from DLS and TEM analyses have slight differences because of the differences in the sample states and methods. As the correlation was studied in our previous work (Haraguchi et al., 2021; Lotya et al., 2013; Yano et al., 2019), *L*_CV_ estimated from DLS analysis was used as a metric of the size distribution. The thickness was measured by AFM and the data were summarized in the histogram (Figures 2B–2F). In the present work, the nanosheets with *N* ≤ 20 are used for the further statistical analysis because the thicker objects have influence on the average values. The average thickness (*t*_ave_) with its standard deviation (*t*_sd_) and average layer number (*N*_ave_) with its standard deviation (*N*_sd_) were *t*_ave_ ± *t*_sd_ = 15.1 ± 7.6 nm and *N*_ave_ ± *N*_sd_ = 9.4 ± 4.7 for the monodispersed (NH_2_-BA)-titanate nanosheets (the number of samples (*n*) = 89, *n* for *N* ≤ 20 (*n*_*N*__≤ 20_) = 67) and *t*_ave_ ± *t*_sd_ = 15.9 ± 15.8 nm and *N*_ave_ ± *N*_sd_ = 4.7 ± 4.6 for the (C_18_-NH_2_)-titanate nanosheets (*n* = 135, *n*_*N*__≤ 20_ = 126) (Figures 2C and 2F). *N*_ave_ with its 95% confidence interval (95% CI) (*N*_ave_ + *N*_CI_, *N*_ave_ − *N*_CI_) was 9.4 (8.2, 10.6) for the monodispersed (NH_2_-BA)-titanate nanosheets and 4.7 (3.9, 5.5) for the polydispersed (C_18_-NH_2_)-titanate nanosheets. As *N*_ave_ with its 95% CI was not overlapped in these two groups, the statistical evaluation supported the significant difference in the layer numbers. An unpaired *t*-test was conducted to validate the differences in the thickness between the monodispersed (NH_2_-BA)-titanate nanosheets and the polydispersed (C_18_-NH_2_)-titanate nanosheets. As p value was less than 0.05 (degree of freedom (df) = 132, *t* = 6.646, p < 0.001), the significant differences in the thickness were verified in the monodispersed and polydispersed conditions. The smaller and larger *L*_CV_ provide the larger and smaller *N*_ave_, respectively. The results imply that the thick and thin nanosheets are selectively obtained under the monodispersed and polydispersed conditions, respectively.

### Selective syntheses of thick and thin nanosheets

**Figure 3:**
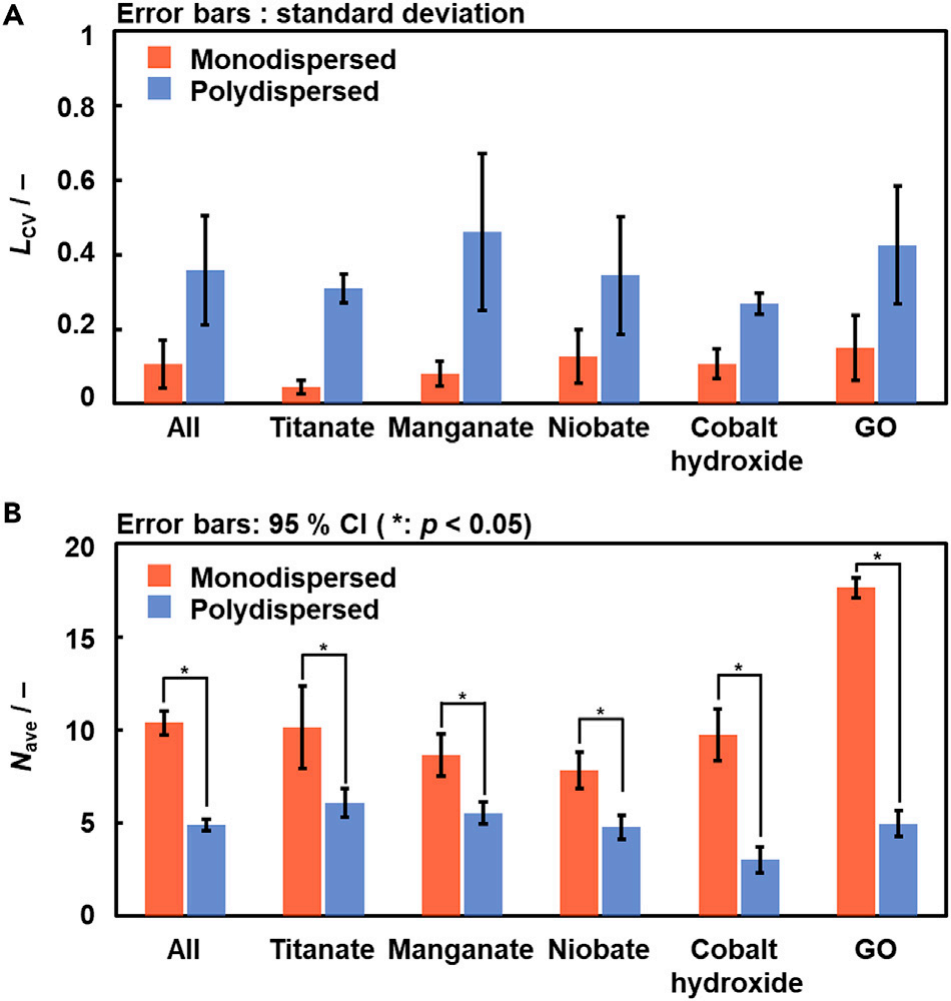
Summary of the measured *L*_CV_ and *N*_ave_ ± *N*_CI_ for the nanosheets derived from the different host layers (titanate, manganate, niobate, cobalt hydroxide, GO) and their average (All) (A) *L*_CV_ and its standard deviation of the monodispersed and polydispersed nanosheets synthesized in the predicted conditions. (B) *N*_ave_ ± *N*_CI_ of the monodispersed and polydispersed nanosheets synthesized in the predicted conditions. The asterisk means the significant difference based on the *t*-test with p < 0.05 (Table S3).

The selective syntheses of the thick and thin exfoliated nanosheets were demonstrated on the assumption of the correlation between the thickness and lateral size distribution (Figure 3). The guest-medium combinations were selected to achieve the thickness-selective syntheses in a limited number of the experiments. The *L*_CV_ prediction model in our previous work assisted the selection of the guest-medium combinations providing larger and smaller *L*_CV_ values for the different host layers (Table 1). In addition, edge-oxidized GO with the layered structures (Wei et al., 2013; Park et al., 2017), a different precursor, was used for the exfoliation (Figure S2 and Table S2). As GO contains no interlayer organic guest, the dispersion media providing the large and small *L*_CV_ values are calculated using the *L*_CV_ prediction model on the assumption that the simplified partial structure of GO was regarded as the guest (Figure S2). Table 1 summarizes the predicted guest-medium combinations for the selective syntheses of the thick and thin nanosheets based on the *L*_CV_ predictor.

**Table 1:** Lateral size distribution and thickness of the exfoliated nanosheets for the different host layered materials

Host layer	Guest	Medium	Measured *L*_CV_/–	Yield/%	*t*_ave_ ± *t*_sd_/nm	*N*_ave_ ± *N*_CI_/–	*n*/–	*n*_*N*__≤ 20_/–
**Monodispersed**								

Titanate	a DEA	Ethanol	0.027	24.2	10.3 ± 6.4	10.1 ± 2.2	91	33
Manganate	b OMe-BA	2-propanol	0.107	8.5	15.1 ± 8.9	8.6 ± 1.1	92	80
Niobate	DEA	h MEA	0.095	78.7	15.9 ± 9.8	7.8 ± 1.0	123	95
Cobalt hydroxide	c HA	2-propanol	0.126	29.0	21.1 ± 12.0	9.7 ± 1.4	103	63
GO	–	1-pentanol	0.071	3.9	5.9 ± 0.7	17.6 ± 0.5	141	60
Average			0.085	16.4	14.3 ± 10.1	10.4 ± 0.6	110	66

**Polydispersed**								

Titanate	d F-BA	Water	0.281	43.4	10.8 ± 7.4	6.1 ± 0.8	125	116
Manganate	e API	Formamide	0.283	7.2	4.5 ± 2.6	5.5 ± 0.6	131	122
Niobate	f CN-BA	Water	0.319	29.9	13.1 ± 10.5	4.8 ± 0.7	131	130
Cobalt hydroxide	g AQ-S	Water	0.279	2.4	8.7 ± 11.1	3.0 ± 0.7	120	116
GO	–	Water	0.496	5.6	1.7 ± 1.0	5.0 ± 0.7	124	69
Average			0.332	17.7	8.4 ± 8.9	4.9 ± 0.3	126	111

a DEA: diethylamine.

b OMeBA: 4-methoxybenzylamine.

c HA: heptanoic acid.

d F-BA: 4-fluorobenzylamine.

e API: 1-(3-aminopropyl)imidazole.

f CN-BA: 4-(aminomethyl)benzonitrile hydrochloride.

g AQ-S: sodium anthraquinone-2-sulfonate monohydrate.

h MEA: 2-methoxyethanol. The source data was in Figures 4 and S3–S6.

The *L*_CV_ values were significantly different for the recommended monodispersed and polydispersed conditions guided by the *L*_CV_ prediction model (Table 1 and Figures 3A, 4A, 4D, and S3–S6). The *t*_ave_ ± *t*_sd_ and *N*_ave_ ± *N*_CI_ for each sample were measured and summarized in Table 1 and Figure 3B. *N*_ave_ (*N*_ave_ + *N*_CI_, *N*_ave_ − *N*_CI_) on the monodispersed conditions was 13.4 (16.4, 10.4) for titanate, 8.6 (9.8, 7.5) for manganate, 11.4 (12.6, 10.2) for niobate, 10.0 (11.6, 8.5) for cobalt hydroxide, and 17.6 (18.1, 17.1) for GO (Figures 3B, 4A–4C, and S3–S6). On the other hand, *N*_ave_ (*N*_ave_ + *N*_CI_, *N*_ave_ − *N*_CI_) on the polydispersed conditions was 6.9 (7.8, 6.0) for titanate, 5.5 (6.1, 5.0) for manganate, 8.3 (9.3, 7.3) for niobate, 3.0 (3.7, 2.3) for cobalt hydroxide, and 6.0 (6.7, 5.3) for GO (Figures 3B, 4D–4F, and S3–S6). *N*_ave_ (*N*_ave_ + *N*_CI_, *N*_ave_ − *N*_CI_) of all the monodispersed and polydispersed nanosheets was 11.8 (12.5, 11.1) and 5.7 (6.0, 5.3), respectively. *N*_ave_ ± *N*_CI_ between the monodispersed and polydispersed conditions had no overlap of the error bar based on 95% CI (Figure 3B). Moreover, *t*-test with p < 0.05 for each host material indicates that the significant differences in the thickness were verified in the monodispersed and polydispersed conditions for all the layered materials (marked with ∗ in Figure 3B and Table S3).

**Figure 4:**
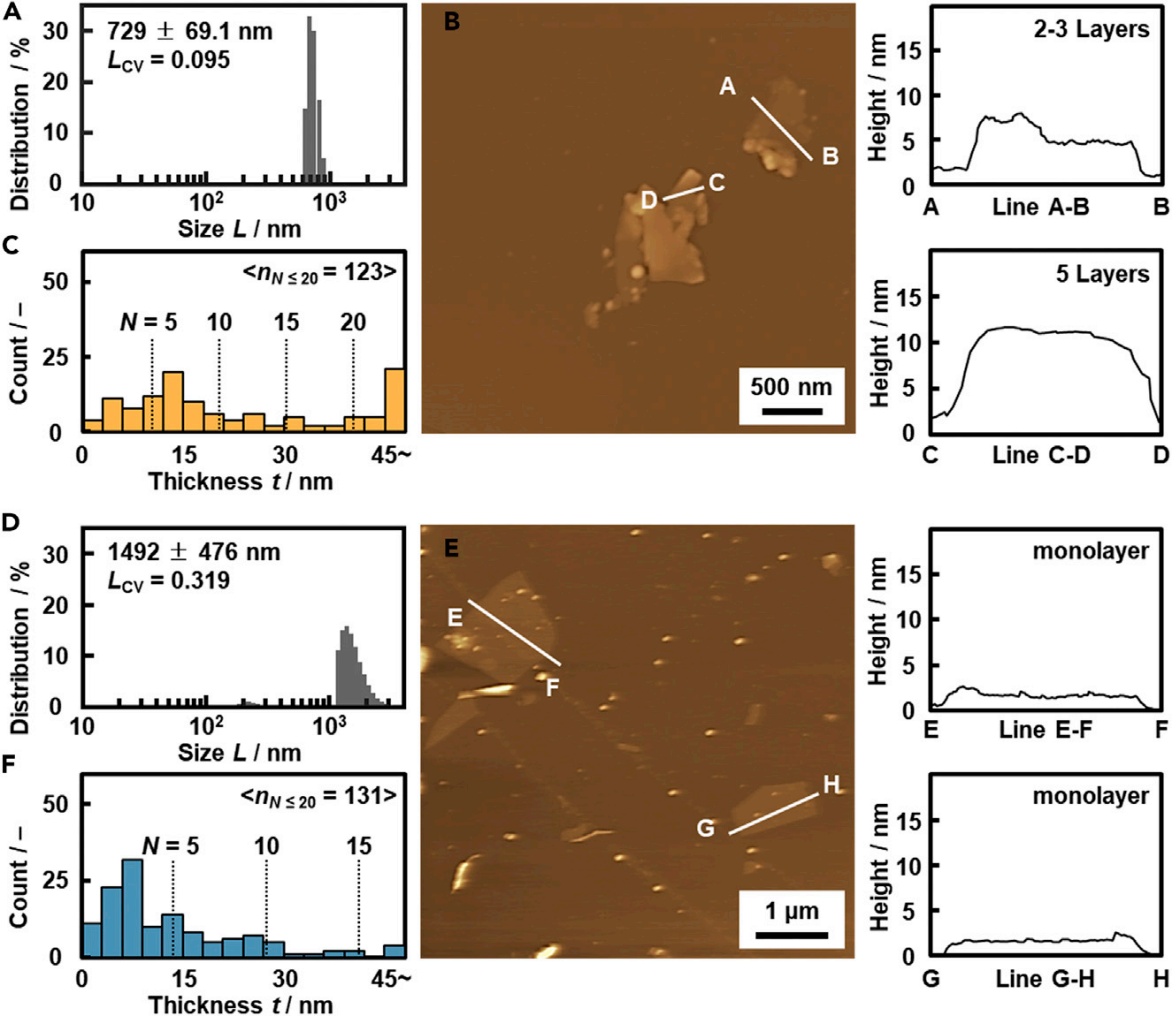
Lateral size distribution and thickness of the (DEA)-niobate nanosheets in 2-methoxyethanol (A–C) and (CN-BA)-niobate nanosheets in water (D–F) (A–D) Lateral size distribution estimated from DLS analyses. (B–E) AFM images and their height profiles. (C–F) Histogram of the thickness based on the AFM images. The same data for the other nanosheets were summarized in Figures S3–S6. Data are represented as mean +/− standard deviation (A) and mean +/− 95% CI (B).

Figure 4 shows the selectively synthesized thick and thin nanosheets based on niobate as a representative case. The layered niobate with the intercalation of diethylamine (DEA) and 4-(aminomethyl)benzonitrile (CN-BA) was exfoliated into the monodispersed and polydispersed nanosheets in 2-methoxyethanol and water, respectively (Table 1). DLS analysis showed *L*_ave_ ± *σ* = 729 ± 69.1 nm and *L*_CV_ = 0.095 for the (DEA)-niobate nanosheets and *L*_ave_ ± *σ* = 1492 ± 476 nm and *L*_CV_ = 0.319 for the (CN-BA)-niobate nanosheets, respectively (Figures 4A and 4D). The monodispersed and polydispersed nanosheets were obtained on the recommended conditions by the *L*_CV_-prediction model. The anisotropic 2D nanostructures were observed on the AFM images (Figures 4B and 4E). The histogram of the thickness indicates formation of the thick (DEA)-niobate nanosheets and thin (CN-BA)-niobate nanosheets (Figures 4C and 4F). The thick and thin nanosheets were similarly observed by AFM on the other host materials (Table 1 and Figures S3–S6). In this way, we initially found the correlation between the thickness and size distribution in the exfoliation of the layered composites based on titanate (Figure 2). The hypothesis was verified not only the other layered composites based on manganate, niobate, and cobalt hydroxide but also GO with the different interlayer interaction (Figures 3, 4, and S3–S6).

## Discussion

**Figure 5:**
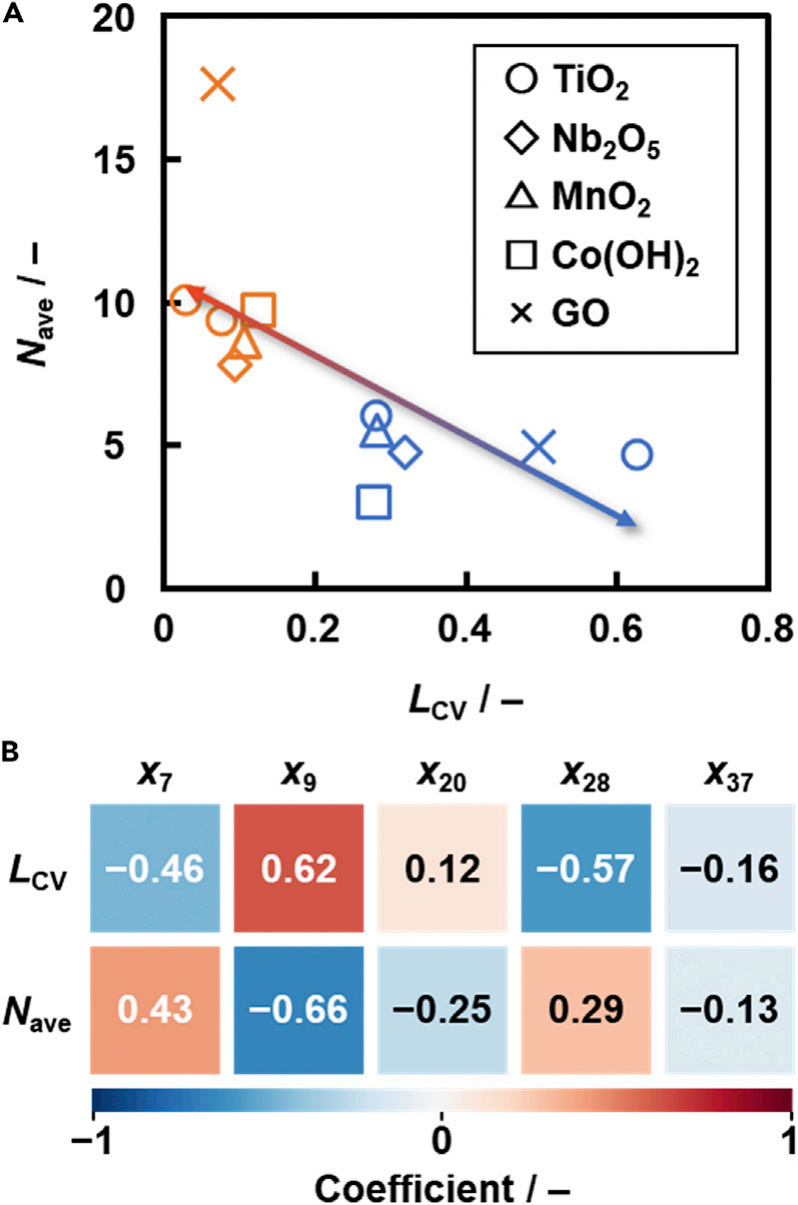
Correlation between *L*_CV_ and *N*_ave_ (A) and their correlation analysis (B) (A) Relationship between the measured *L*_CV_ and *N*_ave_ for the data in Table 1 and Figure 3. (B) Colorimetrically represented correlation coefficients of each descriptor *x*_7_, *x*_9_, *x*_20_*x*_28_, and *x*_37_, as used in the *L*_CV_ prediction model, to *L*_CV_ and *N*_ave_.

Figure 5A summarizes the correlation between the measured *L*_CV_ and *N*_ave_ in Table 1. The thickness-selective syntheses of the nanosheets are achieved by an assistance of the *L*_CV_-prediction model (Equation 1) (Haraguchi et al., 2021), where *x*_7_ is viscosity of the dispersion media, *x*_9_ is surface tension of the dispersion media, *x*_20_ is dipole moment of the guests, *x*_28_ is hydrogen bonding term of Hansen-solubility (similarity) parameter of the guests, *x*_37_ is size of the precursor layered composites, and *L*_CV,pred_ is the predicted *L*_CV_ value.

(Equation 1) $$L_{\text{CV},\text{pred}}=-0.0599x_{7}+0.0802x_{9}+0.0699x_{20}-0.0681x_{28}-0.0623x_{37}+0.266\ldots$$

The descriptors were extracted from *x*_*n*_ (*n* = 1–37) by sparse modeling and our chemical insights in the previous work (Haraguchi et al., 2021). The contribution of the descriptors to *L*_CV,pred_ is compared by the coefficients in (Equation 1), because the descriptors *x*_*n*_ are converted to the normalized frequency distribution such that the mean is 0 and standard deviation is 1. The contribution of each descriptor to *L*_CV_ and *N*_ave_ was analyzed using the correlation coefficients (Figure 5B). The positive and negative of the correlation coefficients to *L*_CV_ were same as those of the coefficients in the *L*_CV_ prediction model (Equation 1). As *L*_CV_ and *N*_ave_ have the negative correlation (Figure 5A), the positive and negative of the correlation coefficients become opposite. The correlation of the descriptors *x*_7_, *x*_9_, *x*_20_, and *x*_28_ to *L*_CV_ was actually opposite in that to *N*_ave_ with the similar correlation coefficients (Figure 5B). In this manner, the statistical analysis also supported the negative correlation between the measured *L*_CV_ and *N*_ave_ in Figure 3.

The thin nanosheets promote frequent random fracture in the lateral direction leading to the polydispersed lateral size. On the other hand, the monodispersed lateral size is achieved by the thick nanosheets without the fracture. The frequency of the exfoliation is related to the thickness and polydispersity. The positive and negative correlations of the descriptors represent the frequency of the exfoliation originating from the types of the guests and dispersion media. The exfoliation proceeds with the intercalation of the dispersion media in the interlayer space containing the guests. The subsequent swelling induces the exfoliation into the nanosheets. The positive correlation of *x*_7_ and negative correlation of *x*_9_ to *N*_ave_ imply that the dispersion media with lower viscosity and higher surface tension promotes the exfoliation of the precursor layered materials into the thinner nanosheets. The dispersion media with low viscosity induce the smooth intercalation in the interlayer space. In addition, the dispersion media with high surface tension are not spread in the interlayer space with wetting but rather clustered to expand the interlayer space with swelling. As shown in Table 1, water and formamide are actually listed as the dispersion media providing the polydispersed thin nanosheets. The guest molecules show the negative correlation of *x*_20_ and positive correlation of *x*_28_ to *N*_ave_. The more polar guests with low hydrogen-bonding ability form the thin nanosheets with the polydispersity. The polar guests accommodate the aforementioned polar dispersion media to promote the swelling and exfoliation. If hydrogen-bonding of the guest-guest and guest-medium is formed in the interlayer space, the dispersion media are not smoothly intercalated. The guests with low hydrogen-bonding ability are preferred to intercalation and swelling with dispersion media. In this manner, the smooth intercalation of the dispersion media in the interlayer space promotes the exfoliation into the thin nanosheets. As the thin nanosheets are easily fractured into the smaller flakes because of the instability, the polydispersed lateral size distribution is achieved in the thin nanosheets. The potential four descriptors of *N*_ave_ can be applied to explore the appropriate layers and/or dispersion media for the selective syntheses of thick and thin nanosheets in a variety of layered materials. As top-down processes include the exfoliation in the vertical direction and fracture in the lateral direction, the monodispersed nanosheets are not easily obtained. On the other hand, a recent paper shows bottom-up synthesis of magnesium hydroxide nanosheets with an assistance of ligands (Muramatsu et al., 2021). If monodispersed and thin nanosheets are required, bottom-up synthesis can be a potential route rather than top-down exfoliation.

In summary, the surface-modified nanosheets were obtained from the layered composites based on transition metal oxides and interlayer guests in organic dispersion media. As the exfoliation behavior is tuned by the host-guest-medium combination, this system is suitable to study the structural and chemical factors related to the thickness. The thickness of the exfoliated nanosheets (*N*_ave_) had a correlation with the lateral size distribution (*L*_CV_). According to the prediction model of the lateral size distribution, the thick and thin nanosheets were selectively obtained on the monodispersed and polydispersed conditions, respectively. Moreover, the selective syntheses based on the correlation were applied to exfoliation of GO with a different type of the interlayer interaction. The statistical study supported the correlation between the thickness and size distribution, i.e. the negative correlation between *L*_CV_ and *N*_ave_. The descriptors of the size-distribution prediction also had correlations with the thickness. The descriptors and their correlations imply the factors related to the thickness based on the chemical insight. The thin nanosheets with the polydispersed lateral size distribution are selectively obtained by the smooth intercalation of the dispersion media in the polar interlayer space through the frequent exfoliation in the vertical direction and fracture in the lateral direction. Our results can be applied to achieve thickness-selective syntheses of a variety of the exfoliated nanosheets using the prediction model of the lateral size distribution.

### Limitations of the study

The model and concept are now applied only to layered transition metal oxides. The further study is needed to generalize the relationship between the thickness and lateral size distribution in a variety of 2D materials.

## STAR★Methods

### Key resources table

**Table:** 

REAGENT or RESOURCE	SOURCE	IDENTIFIER
**Chemicals, peptides, and recombinant proteins**		

Graphene oxide (GO)	Sigma-Aldrich	CAS#796034
Graphite	FUJIFILM Wako Pure Chemical Corp.	CAS#7782-42-5
2-Propanol	Kanto Chemical Co., Inc.	CAS#71-23-8
2-Butanol	Kanto Chemical Co., Inc.	CAS#78-92-2
1-Decanol	Kanto Chemical Co., Inc.	CAS#112-30-1
1-Octanol	Junsei Chemical Co., Ltd.	CAS#11-87-5
1-Pentanol	Tokyo Chemical Industry Co., Ltd.	CAS#71-41-0
Formamide	Kanto Chemical Co., Inc.	CAS#75-12-7
Nitrobenzene	Kanto Chemical Co., Inc.	CAS#98-95-3
Dimethyl sulfoxide (DMSO)	Kanto Chemical Co., Inc.	CAS#67-68-5
1,1,2,2-Tetrabromoethane	Tokyo Chemical Industry Co., Ltd.	CAS#79-27-6

**Software and algorithms**		

Python ver.3.7	Python Software Foundation	https://www.python.org
Gaussian09	Gaussian	https://gaussian.com
Hansen Solubility Parameters in Practice (HSPiP)	HSP and HSPiP	https://www.hansen-solubility.com/
ChemDraw 20.0 and Chem3D 20.0	PerkinElmer	https://www.perkinelmer.com

**Other**		

X-ray diffraction with Cu-Kα radiation (XRD, D8 Advance)	Bruker	https://www.bruker.com/ja/products-and-solutions/diffractometers-and-scattering-systems/x-ray-diffractometers/d8-advance-family/d8-advance.html
Dynamic light scattering (DLS, ELSZ-2000ZS)	Otsuka Electronics	https://www.otsukael.jp/product/detail/productid/92
Atomic force microscopy (AFM, SPM-9700HT)	Shimadzu	https://www.an.shimadzu.co.jp/surface/spm/spm/index.htm

### Resource availability

#### Lead contact

Further information and requests for resources and reagents should be directed to and will be fulfilled by the lead contact, Yuya Oaki (oakiyuya@applc.keio.ac.jp).

#### Materials availability

This study did not generate new unique reagents.

### Method details

#### Exfoliation of the precursor layered composites

Synthesis and characterization of the precursor layered materials were reported in our previous work (Haraguchi et al., 2021; Nakada et al., 2019; Noda et al., 2020; Mizuguchi et al., 2021). The exfoliation experiments of the host-guest-medium combinations in Table 1 were performed in our previous work (Haraguchi et al., 2021). In the present work, their thickness data were newly collected by AFM measurements. In addition, exfoliation of GO was performed. The layered composites were dispersed in organic dispersion for 5 days at 60°C with stirring at 300 rpm (Figure 1A). The dispersion liquids containing the exfoliated nanosheets were obtained after the removal of the unexfoliated bulky particles using filter or cotton depending on the size of the precursors (Haraguchi et al., 2021). The yield was measured on the basis of the weight of the nanosheets collected by membrane filter with the pore size 0.1 μm to the weight of the precursor layered materials (Noda et al., 2020). The dispersion liquid containing the nanosheets was drop-casted on a silicon (Si) substrate. Si substrate was cleaned with immersion in a mixture of hydrochloric acid (HCl) and acetone (1/1 by volume) for 1 h and then in sulfuric acid (H_2_SO_4_) for 1 h. Then, the substrate was rinsed by purified water and then dried with nitrogen flow. The nanosheets were observed by AFM (Shimadzu, SPM-9700HT).

#### Exfoliation of GO and its characterization

Exfoliation of GO was performed in the present work (Figure S2 and Table S2). Edge-oxidized graphene oxide (Aldrich, Graphene oxide powder, 4–10% oxidized) was used as purchased without purification. Graphite (Fujifilm-Wako, Graphite powder, 98%, Particle size (pass 45 μm)) was used as a reference. GO powder (30 mg) was dispersed in 12 cm^3^ of 2-propanol (Kanto, 99.7%), 2-butanol (Kanto, 99.0%), 1-decanol (Kanto, 95.0%), 1-octanol (Junsei, 98.0%), 1-pentanol (TCI, 99.0%), purified water, formamide (Kanto, 98.0%), nitrobenzene (Kanto, 99.5%), dimethyl sulfoxide (DMSO, Kanto, 99.0%), and 1,1,2,2-tetrabromoethane (TCI, 98.0%) for 5 days at 60°C with stirring at 300 rpm. The dispersion liquid was filtered to remove the unexfoliated precursors. The detailed characterization of the GO nanosheets was described in Figure S2 and Table S2. The layered structures were analyzed by X-ray diffraction with Cu-Kα radiation (XRD, Bruker D8 Advance). The particle-size distribution of the nanosheet colloid was measured by dynamic light scattering (DLS, Otsuka Electronics, ELSZ-2000ZS).

### Quantification and statistical analysis

#### Statistic validations

Welch’s *t*-test, namely unpaired *t*-test between two independent groups with the different variances, was carried out using Python (ver. 3.7.4) and excel. The results were used to verify the difference in *N*_ave_ of the nanosheets in the monodispersed and polydispersed conditions. Significance level alpha (*α*) was set at 0.05 in the *t*-test. The null hypothesis was “*N*_ave_ of nanosheets synthesized in the different guest-medium combinations has no significant differences.” The p value indicates the possibility of the null hypothesis is true. The lower p values indicate the significant differences in *N*_ave_. The data used in the *t*-test and the results were summarized in Tables 1 and S3, respectively. In addition, 95% CI was used to verify the difference between two average values. 95% CI means The average value of the population is found to be in the range of the interval with a 95% possibility. The *N*_ave_ and *N*_CI_ were displayed in Figure 3B with “∗” if the p value was smaller than 0.05 in the *t*-test.

#### Data-scientific analysis

The heatmap was prepared by Python to calculate the correlation coefficients (Figure 5B). The positive and negative correlation coefficients were calculated and converted into a red to blue colors, respectively. The data for 12 thickness measurements in Figures 2, 4, and S3–S6 were set as the objective variable. The descriptors of the *L*_CV_-prediction model, namely *x*_7_, *x*_9_, *x*_20_, *x*_28_, and *x*_37_, were used as the explanatory variables.

## Data Availability

Data reported in this paper will be shared by the lead contact upon request. This paper does not report original code. Any additional information required to reanalyze the data reported in this paper is available from the lead contact upon request.
