# Supplementary material for: Selective syntheses of thick and thin nanosheets based on correlation between thickness and lateral-size distribution
Source: iScience. 2022 Aug 24;25(9):104933. doi: 10.1016/j.isci.2022.104933 (PMC9463570; doi:10.1016/j.isci.2022.104933)
Supplement: Document S1. Figures S1–S6 and Tables S1–S3 [file mmc1.pdf]

iScience, Volume *25*

Supplemental information

Selective syntheses of thick and thin nanosheets based on correlation between thickness and lateral-size distribution

Yuri Haraguchi, Hiroaki Imai, and Yuya Oaki

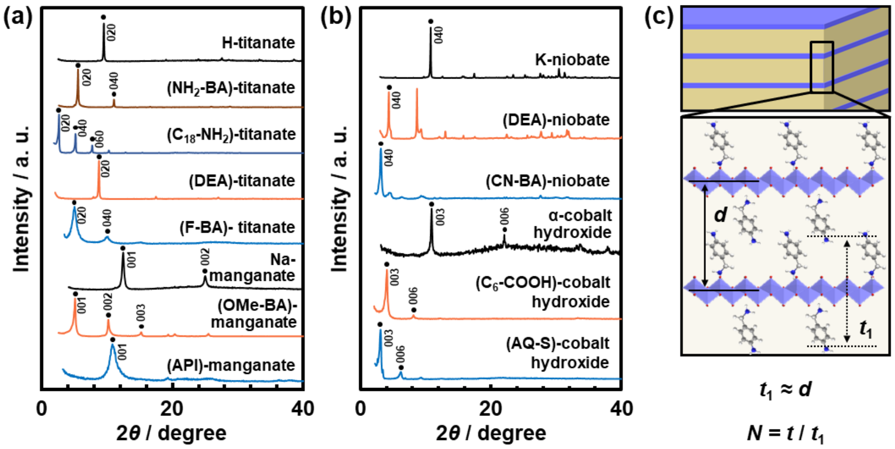

Figure S1. X-ray diffraction (XRD) patterns (a,b) and schematic illustration (c) of the precursor layered materials. (a) XRD patterns of layered titanate and manganate without and with the intercalation of the guests. (b) XRD patterns of layered niobate and cobaltate without and with the intercalation of the guests. (c) Schematic illustration of the layered structures consisting of the host and interlayer guests for definition of the layer number (*N*). Related to Figure 2 and Table 1.

The abbreviations of the guests in the panels (a,b) were referred to Table 1 in the main text. These guests were selected on the basis of the L_{CV}-prediction model. The detailed structure characterization of these precursor layered materials was reported in our previous works. The interlayer distance (*d*) measured by XRD (*d*) was summarized in Table S1. In the present work, the layer number (*N*) was calculated on the basis of the *d* values by the following procedure. The thickness of nanosheets (*t*) was measured by AFM (Figure 4 and Figures S3–S6). The interlayer distance (*d*) was estimated from the XRD patterns (Figure S1a,b and Table S1). Here the thickness of the monolayer (t_1) is approximated to *d* (Figure S1c), although the tilted angle of the guest molecules is slightly different for the monolayered state. *N* was defined as t / t_1 and then N_{ave} was calculated. As for niobate nanosheets, t_1 was defined as the thickness of the bilayered structure. The layered niobate ($K_4Nb_6O_{17}\cdot 3H_2O$) has the two different types of the interlayer space, namely the interlayer space I and II. The previous study showed that the guest organic molecules are easily intercalated in the interlayer I compared with the interlayer II. According to the previous work, the guest is only intercalated in the interlayer I when *x* is smaller than 3.2. The ratio of the intercalated guests, namely *x* in (guest)$_x$K$_{4-x}$Nb$_6$O$_{17}$, was calculated using TG analysis in our previous report, such as x = 1.314 for (DEA)-niobate and x = 2.222 for (DEA)-niobate. Therefore, the thickness of the monolayer was calculated on the assumption that the exfoliation proceeded only from the interlayer I in the present work.

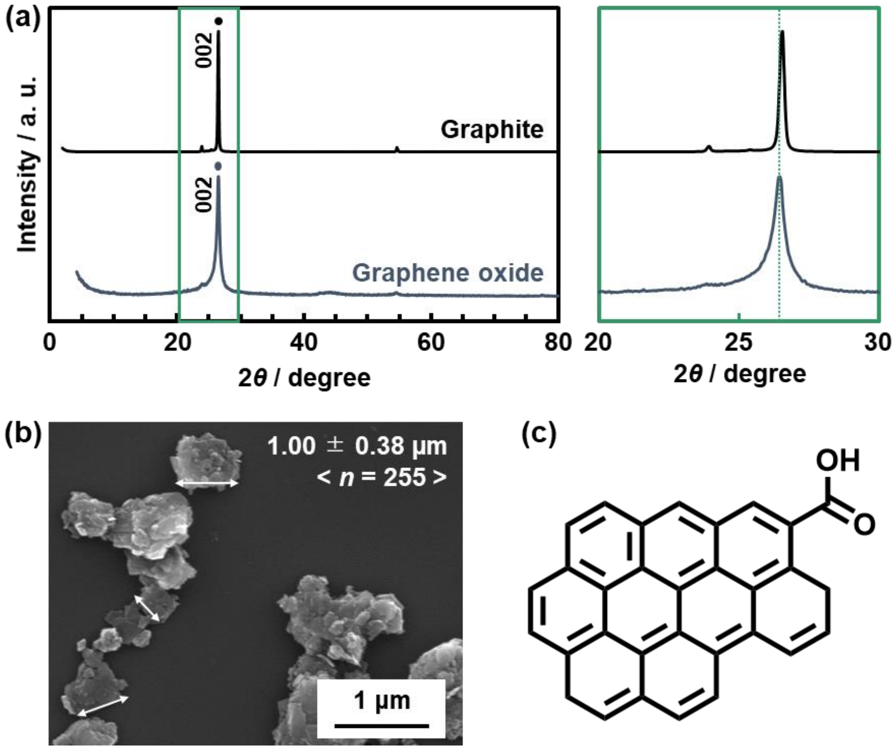

Figure S2. Structural characterization of GO as the precursor layered material. (a) XRD pattern and its magnified one of GO and reference graphite. (b) Scanning electron microscopy (SEM) image. (c) Partial unit structure estimated from CHN elemental analysis. Related to Figure 2 and Table 1.

The purchased sample was edge-oxidized graphene oxide (Aldrich, 4–10 % edge-oxidized). The XRD patterns show that the interlayer distance was 0.335 nm for graphite and 0.336 nm for GO (Figure S2a). In addition, the broadening of the 002 peak indicates the lowering the crystallinity because of the edge oxidation (the magnified pattern in Figure S2a), as reported in the previous works. The average particle size was 1.00 ± 0.38 μm. The smaller unit flakes were observed in the GO particles (Figure S2b). The composition of GO was C : H : N : O (the other component) = 91.1: 0.6: 0.7: 7.6 from CHN elemental analysis. The reason why N is contained in the GO sample is unclear. The partial unit structure was depicted on the basis of the composition of C, H, and O (Figure S2a). The estimated structure was used for calculation of the descriptors in the L_{CV}-prediction model.

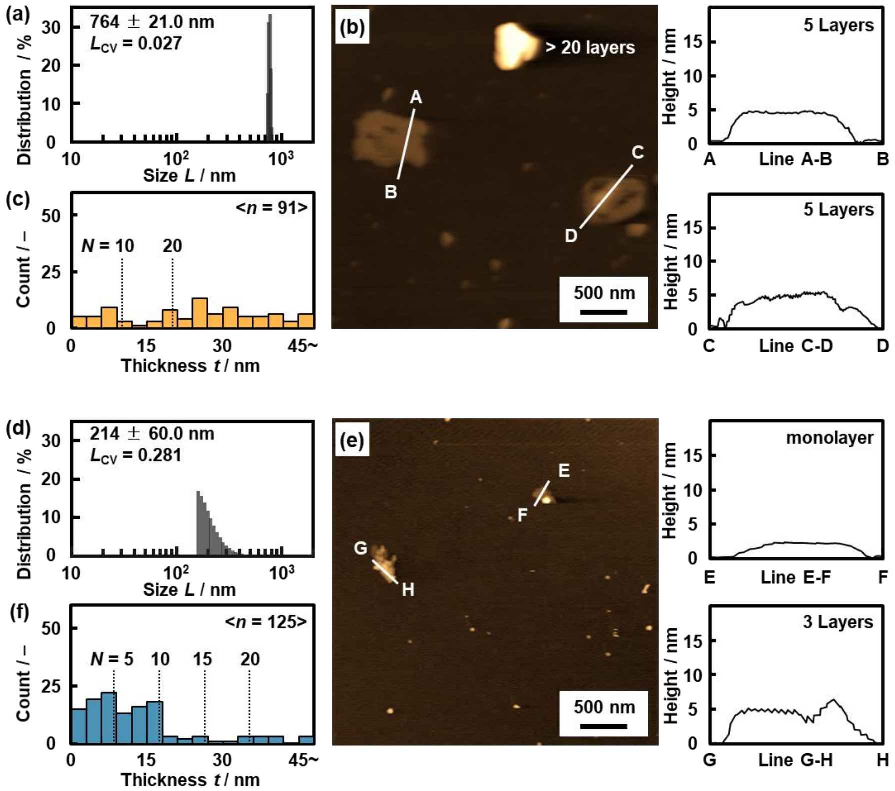

Figure S3. Exfoliated nanosheets of thick (DEA)-titanate nanosheets in ethanol with monodispersity (a–c) and (F-BA)-titanate nanosheets in water with polydispersity (d–f). (a,d) DLS charts. (b,e) AFM images and their height profiles. (c,f) Histogram of the thickness based on the AFM images. Related to Figure 3 and Table 1.

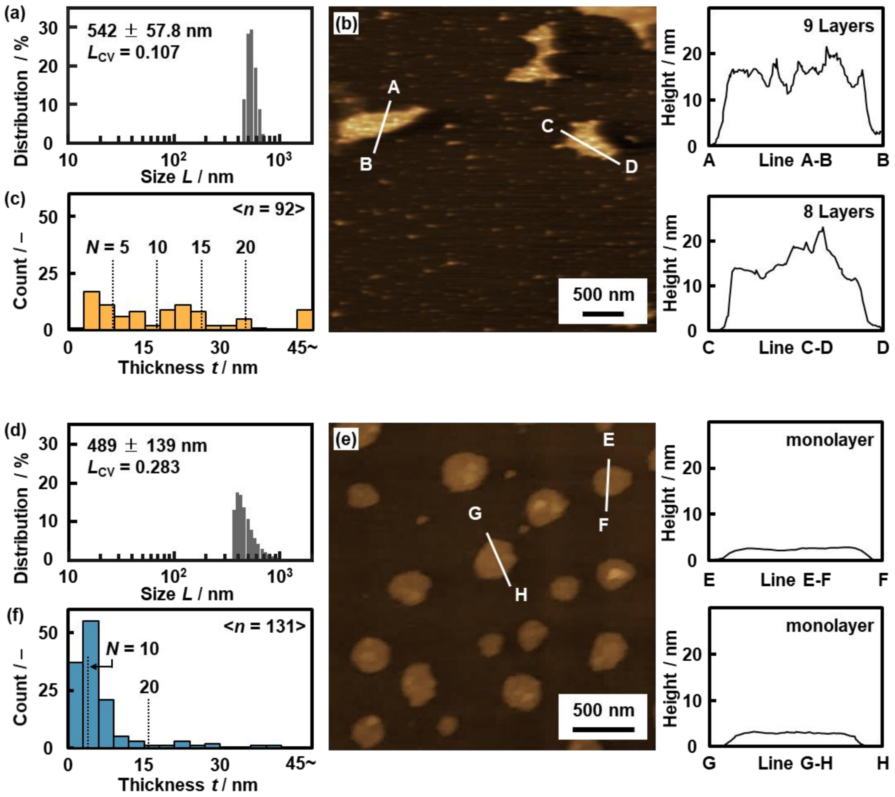

Figure S4. Exfoliated nanosheets of thick (OMe-BA)-manganate nanosheets in 2-propanl with monodispersity (a–c) and thin (API)-manganate nanosheets in formamide with polydispersity (d–f). (a,d) DLS charts. (b,e) AFM images and their height profiles. (c,f) Histogram of the thickness based on the AFM images. Related to Figure 3 and Table 1.

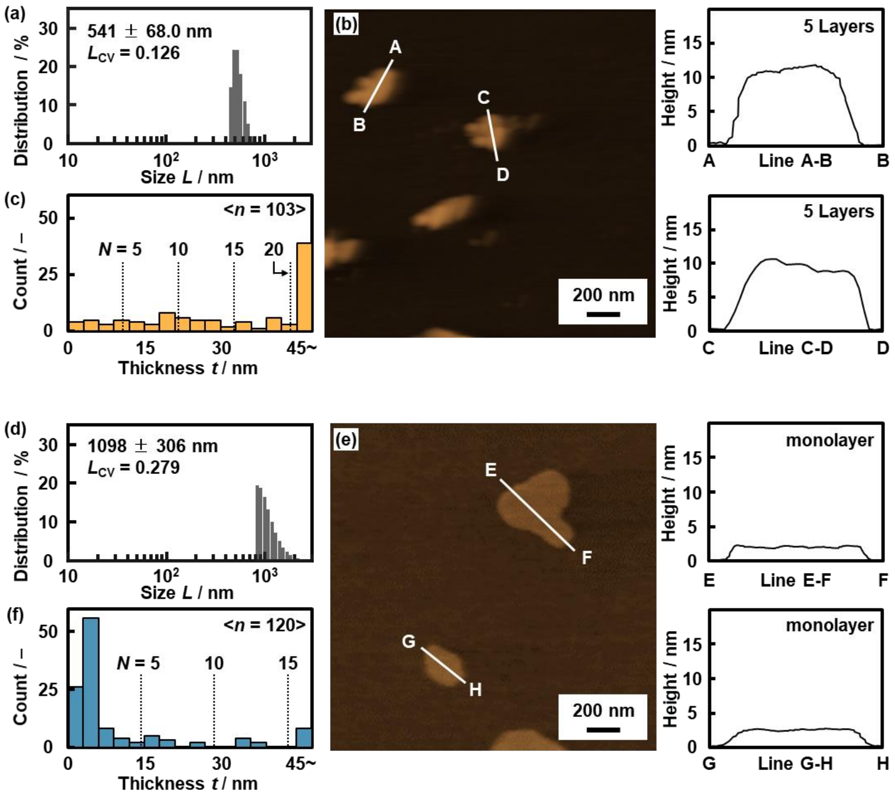

Figure S5. Exfoliated nanosheets of thick (HA)-cobalt hydroxide nanosheets in 2-propanol with monodispersity (a–c) and thin (AQ-S)-cobalt hydroxide nanosheets in water with polydispersity (d–f). (a,d) DLS charts. (b,e) AFM images and their height profiles. (c,f) Histogram of the thickness based on the AFM images. Related to Figure 3 and Table 1.

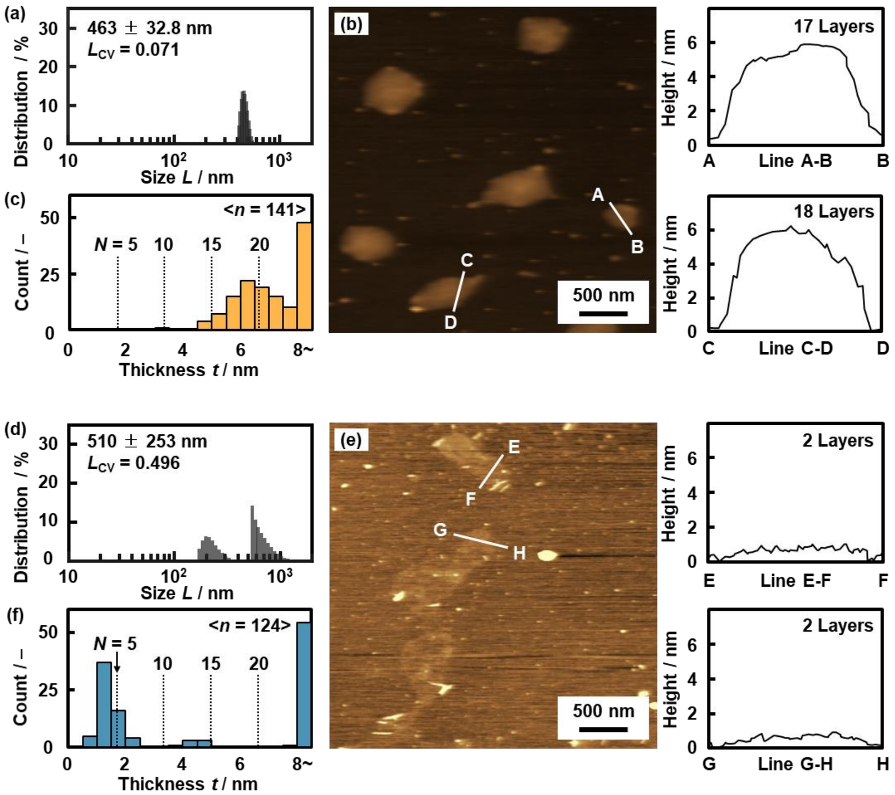

Figure S6. Exfoliated nanosheets of thick GO in 1-pentanol with monodispersity (a–c) and thin GO in water with polydispersity (d–f). (a,d) DLS charts. (b,e) AFM images and their height profiles. (c,f) Histogram of the thickness based on the AFM images. Related to Figure 3 and Table 1.

Table S1. Summary for *d* of the precursor layered composites used in the present work. Related to Figure 2 and Table 1.

Titanate	*d*	Manganate	*d*	Niobate	*d*	Cobalt hydroxide	*d*		*d*
Guest	d_{020} / nm	Guest	d_{001} / nm	Guest	d_{040} / nm	Guest	d_{003} / nm		d_{002} / nm
NH_2-BA	1.602	OMe-BA	1.746	DEA	2.026	C_6-COOH	2.176	Graphite	0.335
C_{18}-NH_2	3.390	API	0.821	CN-BA	2.754	AQ-S	2.889	GO	0.336
DEA	1.013								
F-BA	1.781								

Table S2. Top and bottom five conditions for selective syntheses of the monodispersed (M1–5) and polydispersed (P1–5) GO nanosheets, respectively. Related to Figure 3 and Table 1.

Monodispersed					Polydispersed				
Rank	Medium	Predicted L_{CV} / –	Measured L_{CV} / –	Yield / %	Rank	Medium	Predicted L_{CV} / –	Measured L_{CV} / –	Yield / %
M1	2-propanol	0.305	0.262	2.2	P1	water	0.716	0.496	5.6
M2	2-butanol	0.306	0.051	2.7	P2	formamide	0.587	0.346	23.4
M3	1-decanol	0.307	0.175	28.4	P3	nitrobenzene	0.495	0.335	19
M4	1-octanol	0.315	0.189	23.4	P4	DMSO	0.49	0.669	20.9
M5	1-pentanol	0.318	0.071	3.9	P5	1,1,2,2-tetrabromoethane	0.481	0.281	7.4
Average		0.310	0.150	12.1	Average		0.554	0.425	15.3
Standard deviation		0.006	0.088	12.7	Standard deviation		0.100	0.158	8.2

GO was exfoliated into the nanosheets in the selected organic dispersion media (Table S2). According to our previous work, the prediction model of the size distribution (L_{CV}) is comprised of the five descriptors about the physicochemical parameters of the guest and medium and lateral size of the host ((Eq. 1) in the main text). Here the GO layer itself was regarded as the guest because the interlayer guest molecules were not introduced. The physicochemical parameters of GO were calculated on the assumption that GO is the guest of the layered composites. The dispersion media providing the monodispersed and polydispersed nanosheets were selected from 95 candidates using the L_{CV}-prediction model (Table S2).

Table S3. Summary of statistical validations for the *t*-test. Related to Figure 3 and Table 1

	df	*t*	*p*
All	486	15.15	< 0.001*
titanate	40	3.50	0.00114*
niobate	173	5.15	< 0.001*
manganate	120	4.86	< 0.001*
cobalt hydroxide	95	8.56	< 0.001*
GO	121	28.97	< 0.001*

*: $p < 0.05$, df: degree of freedom.

Weltch's *t*-test shows the statistical difference in N_{ave} of nanosheets obtained in the monodispersed and polydispersed conditions for each host material (Figure 3b). The comparisons were conducted on N_{ave} in the two groups, such as the predicted monodispersed and polydisperse conditions with the different guest-medium combinations. In Table S3, the *p* values were smaller than 0.001 (< 0.001). The *p* values in *t*-test were less than 0.05 for each host material. On the basis of these results, we concluded that the significant difference between N_{ave} of the nanosheets was observed in the predicted monodispersed and polydispersed conditions (Figure 3b).
